# Hydroxyl radical mediated damage of proteins in low oxygen solution investigated using X-ray footprinting mass spectrometry

**DOI:** 10.1107/S1600577521004744

**Published:** 2021-07-20

**Authors:** Line G. Kristensen, James M. Holton, Behzad Rad, Yan Chen, Christopher J. Petzold, Sayan Gupta, Corie Y. Ralston

**Affiliations:** a Molecular Biophysics and Integrated Bioimaging Division, Lawrence Berkeley National Laboratory, 1 Cyclotron Road, Berkeley, CA 94720, USA; b Molecular Foundry Division, Lawrence Berkeley National Laboratory, 1 Cyclotron Road, Berkeley, CA 94720, USA; c Biological Systems and Engineering Division, Lawrence Berkeley National Laboratory, 1 Cyclotron Road, Berkeley, CA 94720, USA

**Keywords:** X-ray footprinting mass spectrometry (XFMS), hydroxyl radical, radiation damage

## Abstract

In the method of X-ray footprinting mass spectrometry (XFMS), proteins at micromolar concentration in solution are irradiated with a broadband X-ray source, and the resulting hydroxyl radical modifications are characterized using liquid chromatography mass spectrometry to determine sites of solvent accessibility. These data are used to infer structural changes in proteins upon interaction with other proteins, folding, or ligand binding. XFMS is typically performed under aerobic conditions; dissolved molecular oxygen in solution is necessary in many, if not all, the hydroxyl radical modifications that are generally reported. In this study we investigated the result of X-ray induced modifications to three different proteins under aerobic versus low oxygen conditions, and correlated the extent of damage with dose calculations. We observed a concentration-dependent protecting effect at higher protein concentration for a given X-ray dose. For the typical doses used in XFMS experiments there was minimal X-ray induced aggregation and fragmentation, but for higher doses we observed formation of covalent higher molecular weight oligomers, as well as fragmentation, which was affected by the amount of dissolved oxygen in solution. The higher molecular weight products in the form of dimers, trimers, and tetramers were present in all sample preparations, and, upon X-ray irradiation, these oligomers became non-reducible as seen in SDS-PAGE. The results provide an important contribution to the large body of X-ray radiation damage literature in structural biology research, and will specifically help inform the future planning of XFMS, and well as X-ray crystallography and small-angle X-ray scattering experiments.

## Abstract 2

The method of X-ray footprinting mass spectrometry was used to investigate the effect of X-ray irradiation on various proteins in solution under both fully aerated and low dissolved oxygen conditions, and as a function of protein concentration.

## 1. Introduction   

X-ray footprinting mass spectrometry (XFMS) is a solvent accessibility-based method that is used to obtain structural information on biological macromolecules in solution. In the general implementation of this method, a protein or protein complex in a water-based solution is exposed to an X-ray source, and the radiolysis of the water produces hydroxyl radicals (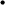
OH). The hydroxyl radicals modify proteins and/or cleave nucleic acid in solvent-accessible regions. Protein modifications are generally investigated using standard bottom-up liquid chromatography mass spectrometry (LC-MS) analysis (Gupta *et al.*, 2007 ▸). The XFMS method yields information on water positions within or at the surface of the macromolecule, which is then used to infer structural information, such as sites of water occlusion during protein–protein interactions (Gupta *et al.*, 2016 ▸). While the use of solvent accessibility methods to gain information on structural features in proteins and nucleic acids has a long history, the use of X-rays to generate hydroxyl radicals for the method is relatively new (Sclavi *et al.*, 1998 ▸). XFMS offers the advantages of both accessing a shorter timescale of structural events and limiting secondary oxidation reactions by delivering a fast, high-intensity burst of radiation. Another advantage of 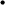
OH footprinting over methods such as DNase footprinting or chemical footprinting methods is the small size of the hydroxyl radical, which permits finer structural features to be resolved in nucleic acids or proteins. Two national XFMS synchrotron beamlines are now in operation (Asuru *et al.*, 2019 ▸; Gupta *et al.*, 2014 ▸), and XFMS has several experimental aspects that distinguish it from the more well known X-ray protein structural methods, such as macromolecular crystallography (MX) and small angle X-ray scattering (SAXS).

First, protein concentration in the XFMS experiment is typically maintained between 1 and 10 µ*M*, in contrast with the higher concentrations necessary in SAXS experiments (Skou *et al.*, 2014 ▸). This ensures that the radiation dose is mainly absorbed by the water, present at 55 *M*, and other buffer components, typically maintained in the 10–50 m*M* concentration range. Radiation damage to the protein is therefore assumed to be mediated by the products of water radiolysis, instead of due to direct interaction with X-rays. This has made calculation of dose effect on protein damage challenging, since for an identical radiation dose [energy lost per unit mass, J kg^−1^ = Gy (grays)] to the solution, damage to the protein might vary by orders of magnitude depending on the level of scavengers present in the solution, because of the variability in 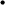
OH radical availability. In general, X-ray exposure in the XFMS experiment is carefully controlled in order to minimize overall oxidative damage to the protein. The ideal time of exposure is determined by the linearity of the so-called ‘dose response’ curve, which is a measure of the modification of a residue as a function of hydroxyl radical concentration. In practice, the dose response curve in an XFMS experiment is a plot of fraction of unmodified product for a given residue as a function of X-ray exposure time. Depending on the source of X-ray radiation, buffer and added scavengers, exposure times can be as low as microseconds or as high as milliseconds.

Second, because the rates of interaction of hydroxyl radical with protein are generally an order of magnitude higher than that of solvated electrons or other radiolysis products, including hydrogen peroxide and superoxide (Davies, 2016 ▸), only 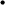
OH modifications to the protein are characterized in subsequent LC-MS analysis. However, other radiolysis products, such as hydrogen peroxide, can persist in solution long after the X-ray exposure is complete, and so, in practice, in the XFMS experiment, irradiated solutions are quenched with a scavenger molecule such as me­thio­nine amide or frozen immediately after exposure to the X-ray beam to limit secondary oxidation damage unrelated to hydroxyl radical damage.

Third, XFMS experiments are generally conducted in the solution state using aerated samples at room temperature. Very few footprinting studies have been conducted using low oxygen solutions, though some studies of N_2_O-purged samples have been conducted, since N_2_O converts electrons to hydroxyl radicals and can be used to increase the hydroxyl radical to protein ratio (Gupta *et al.*, 2014 ▸; Watson *et al.*, 2009 ▸). Dissolved oxygen in solution is assumed to be necessary for many, if not all, the 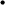
OH modification products in the XFMS experiment (Xu & Chance, 2007 ▸). The generation of solvated electrons during radiolysis further complicates predictions of radiation damage to proteins under anaerobic conditions, since these electrons may also attack protein backbone and/or side-chains, and compete for dissolved oxygen (Hawkins & Davies, 2001 ▸). Despite the challenge of working with anaerobic solutions, the study of radiation-mediated interactions with proteins under anaerobic conditions is important as it is relevant to the development of *in vivo* hydroxyl radical footprinting.

XFMS is a potentially unique and powerful method to determine protein–protein interactions, dynamics and structure within whole cell environments, yet the concentration of dissolved oxygen in cells can be very low depending on the cell type and location within the cell (Ebbesen *et al.*, 2004 ▸). Despite this challenge, some *in vivo* hydroxyl radical footprinting studies have been successfully conducted. In particular, hydroxyl radical cleavage of nucleic acid, in contrast to hydroxyl radical modification of protein, does not require the presence of oxygen, and XFMS has been successfully used to determine the dynamics of ribosome assembly within live *E. coli* cells (Hulscher *et al.*, 2016 ▸). In addition, the hydroxyl radical footprinting method fast photochemical oxidation of proteins (FPOP) has been successfully demonstrated to yield solvent accessibility information on proteins within live Vero cells (Espino *et al.*, 2015 ▸). In the FPOP method, UV irradiation of hydrogen peroxide generates 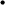
OH; in these whole cell studies, the H_2_O_2_ also served to elicit a cellular response to generate oxygen internally during the experiment. In this study, we used XFMS to investigate the effect of dissolved oxygen on X-ray radiation-induced damage of proteins in solution at room temperature, characterizing the type and extent of fragmentation and oxidation products, and correlating calculated dose in gray (= J kg^−1^) with extent of damage.

## 2. Materials and methods   

### 2.1. Sample preparation   

Equine heart cytochrome *c* (C2506), lysozyme from chicken egg white (L6876), bovine serum albumin (A7638) and equine skeletal muscle myoglobin (M0630) were purchased in lyophilized form from Sigma-Aldrich. Samples were prepared to 2, 5, 20 or 200 µ*M* concentration by dissolving in 10 m*M* phosphate buffer at pH 7.3, and divided into 1 ml aliquots. Low oxygen samples were further prepared by cycling into a nitro­gen environment in a Coy Anaerobic Chamber and allowed to equilibrate overnight. Dissolved oxygen levels as measured using a Milwaukee MW600 sensor inside the chamber showed that, upon first entering the anaerobic chamber, samples contained 14–17 mg L^−1^ dissolved oxygen, and after equilibrating overnight in the chamber, contained 1–3 mg L^−1^. The low oxygen protein samples were drawn up one at a time into a gas-tight glass luer-lock Hamilton syringe, wrapped with parafilm, double-bagged inside the anaerobic chamber, and brought immediately to beamline 3.2.1 for data collection.

### 2.2. XFMS experiment   

X-ray irradiation was performed at ALS beamline 3.2.1, a 1.3 T bending-magnet beamline with critical energy of 3100 eV and beam size of ∼10 mm × 100 mm, and no monochromator or focusing optics in the beam path. The beam was apertured using 5 mm-thick Pb to 2 mm × 4 mm at the beam-pipe exit window in the hutch, and the aperture was aligned to the beam center using a photodiode. Prior to irradiation of protein samples, beam flux was characterized using 5 µ*M* Alexa 488 fluorescence dye (Thermo Fisher Scientific) in 10 m*M* phosphate buffer as previously described (Gupta *et al.*, 2007 ▸). Protein samples pre-loaded into syringes were placed in the syringe pump capillary flow X-ray footprinting instrument (Gupta *et al.*, 2014 ▸). Irradiation time was varied between 0 and 200 ms as determined by flow speed (Gupta *et al.*, 2020 ▸). During the course of a complete XFMS dose response collection, which was approximately 5 minutes, oxygen from the air likely began diffusing back through the exit end of the 200 µm-diameter capillary tubing and into the syringe; therefore, exposure times for the anaerobic samples were collected only up to 100 ms. Irradiated samples were immediately placed on dry ice or placed in a −80°C freezer to limit further oxidation before digestion and LC-MS and/or matrix-assisted laser desorption/ionization (MALDI) analysis.

### 2.3. MALDI, LC-MS, and SDS-PAGE   

Protein samples were prepared for MALDI analysis by spotting onto Bruker MSP 96 target polished steel plates with α-cyano-4-hy­droxy­cinnamic acid matrix in a 1:2 protein-to-matrix ratio. A Bruker AB Sciex TF4800 TOF-TOF mass spectrometer was used to collect MALDI spectra. Protein samples for LC-MS analysis were reduced with 5 m*M* DTT at 60°C for 30 min followed by 15 m*M* iodo­acetamide alkyl­ation for 30 min at room temperature in the dark. Samples were desalted and buffer exchanged into 10 m*M* ammonium bicarbonate using 7 K MWCO, 0.5 ml Zeba spin desalting columns (Thermo Fisher) according to the manufacturer’s instructions. The desalted protein samples were digested overnight at 37°C with mass spectrometry grade Trypsin/Lys-C protease mix (Promega) at a 1:20 (*w*/*w*) protease:protein ratio. Cyt *c*, lysozyme, and myoglobin showed 96%, 88%, and 92% sequence coverage, respectively, for both aerated and low oxygen sample conditions. LC-MS/MS analysis of peptides was conducted on an Orbitrap Exploris 480 mass spectrometer (Thermo Fisher Scientific) coupled to an Agilent 1290 UHPLC system (Agilent Technologies, Santa Clara, CA, USA). Peptides were separated on a InfinityLab Poroshell 120 EC-C18 column (2.1 mm × 100 mm, 1.9 µm particle size, operated at 60°C) at a 0.400 ml min^−1^ flow rate and eluted with the following gradient: initial condition was 98% solvent A (0.1% formic acid) and 2% solvent B (99.9% aceto­nitrile, 0.1% formic acid). Solvent B was increased to 10% over 1.5 min, and then increased to 35% over 10 min, then up to 80% over 0.5 min, and held for 1.5 min at a flow rate of 0.6 ml min^−1^, followed by a ramp back down to 2% B over 0.5 min where it was held for re-equilibrating the column to original conditions. The mass spectrometer was operated with the following settings: full scan Orbitrap resolution at 60000; AGC target at 3.0 × 10^6^; maximum injection time after 60 ms; top ten intense ions were isolated for HCD fragmentation per MS scan with collision energy set to 30% and intensity threshold at 5.0 × 10^3^; dynamic exclusion duration set at 10 s; data-dependent MS2 scan Orbitrap resolution at 15000; AGC target at 1.0 × 10^5^; and maximum injection time after 50 ms. Protein samples were prepared for SDS-PAGE by heating at 95°C for 5 min in Laemmli sample buffer (Bio-Rad) containing 2-mercapto­ethanol at a final concentration of 2.5% (*v*/*v*). SDS-PAGE was performed using 4–20% Criterion TGX gels (Bio-Rad) in Tris/Glycine/SDS running buffer (Bio-Rad). The protein loading amount was 3 µg per well (1.4 µg per well for 5 µ*M* cytochrome *c*). Gels were stained with Imperial protein stain (Thermo Fisher).

### 2.4. Dose calculations   

Dose calculations were completed using *RADDOSE-3D* (Bury *et al.*, 2018 ▸) for materials in the 3.2.1 beampath as listed: 375 µm total Be window thickness, 25.4 µm Al, 38.1 mm air gap, 10 µm capillary coating (carbon at 0.9 g cm^−3^), 80 µm capillary thickness (SiO_2_ at 2.203 g cm^−3^), and 200 µm sample thickness (H_2_O at 1 g cm^−3^). The beam profile for *RADDOSE-3D* was input as a top-hat with rectangular collimation of 200 µm × 200 µm. Because protein concentrations in an XFMS experiment are on the order of micromolar, and water is present at 55 *M*, all calculations assume that the absorbed radiation dose is due entirely to the water, and no direct interaction of X-rays with protein is considered. The Center for X-ray Optics radiation calculator (https://henke.lbl.gov/optical_constants; Henke *et al.*, 1993 ▸) was used to calculate the photon spectrum, given a distance of 17.25 m from the source point to the sample.

### 2.5. Analysis of LC-MS data   

LC-MS data were analyzed using the *Byos v3.11-1* software platform (Protein Metrics Inc.) which incorporates the Byonic MS/MS search engine and a customized Oxidative Footprinting workflow for the identification and quantification of protein modifications. In addition, certain modifications were manually analyzed using *Xcalibur* (Thermo Scientific). Hydroxyl radical-mediated mass modifications searched for were the typical +14, +16, +32, +48, −30 Da footprinting probes (Xu & Chance, 2007 ▸) as well as the rare modifications with −2, −16, and +30 Da mass shifts. The quantification of modifications is based on the extracted ion chromatogram peak area of the modified and unmodified peptides. The calculation of the fraction of unmodified peptide was in accordance with the established method (Gupta *et al.*, 2016 ▸). The fraction of unmodified peptide as a function of exposure time was used to plot the dose response of the residue(s). Dose responses were plotted in *Origin* 2019b (OriginLab) and curve fitted using a single exponential fit to provide site-specific modification rate constants, *k* (s^−1^).

## 3. Results and discussion   

### 3.1. Dependence of radiation damage on protein concentration   

**Figure 1:**
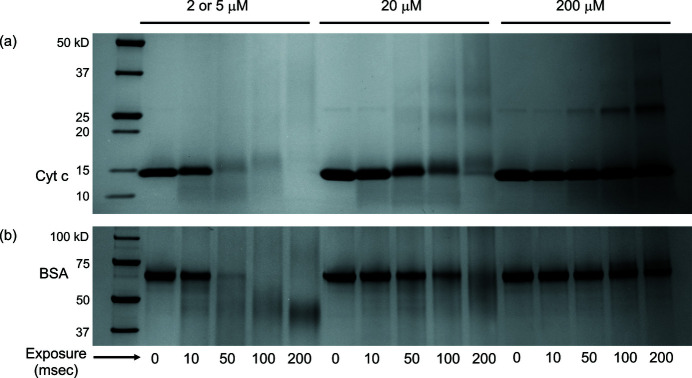
Concentration dependence of X-ray damage. SDS-PAGE of cyt *c* (*a*) and BSA (*b*) after X-ray irradiation of 0–200 ms and for different protein concentrations. BSA concentrations were 2, 20, and 200 µ*M*, while cyt *c* concentrations were 5, 20, and 200 µ*M*. For reference, the calculated absorbed radiation dose by the buffer was 162 Gy for a 10 ms X-ray exposure and 3240 Gy for a 200 ms exposure.

Cyt *c* and BSA proteins in phosphate buffer were first irradiated under standard aerobic conditions in concentrations of 2 (or 5), 20 and 200 µ*M* in order to investigate the effect of protein concentration on rates of oxidative modification. Reducing SDS-PAGE results (Fig. 1 ▸) show a clear protective effect of protein concentration. To control for the effect of capillary flow on sample damage, protein samples of the same concentration and for the same exposure times were irradiated using a static tube holder and X-ray shutter and gave qualitatively similar results (data not shown). The ‘dilution effect’ in which radiation damage is increased for lower protein concentrations has been previously reported in SAXS studies (Kuwamoto *et al.*, 2004 ▸; Hopkins & Thorne, 2016 ▸; Stachowski *et al.*, 2020 ▸) and can be understood in terms of the relative amounts of protein to oxidative attackers (primarily 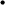
OH and oxygen). In the case of XFMS, for instance, for a given radiation dose to the solution, a consistent steady-state concentration of 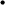
OH will be produced during the time that the X-ray beam is impinging on the solution. These radicals diffuse only on the order of several molecular lengths before recombining with other radiolysis products because of their high reactivity (Janik *et al.*, 2007 ▸; Attri *et al.*, 2015 ▸). As the concentration of the protein decreases while the 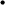
OH concentration remains the same, the ratio of 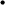
OH molecule (and oxygen molecule) to protein molecule increases. It is interesting that the fraction remaining as a function of X-ray exposure for both proteins studied here, although of very different molecular weights, appear qualitatively similar. This suggests that the dilution effect could be independent of protein size within a certain range. However, many factors play a role in overall oxidation, including surface hydration, surface topography, level of disorder, and surface residue composition, and so the relationship between the size of the protein and amount of oxidation for a given X-ray dose is complicated. A more comprehensive study using a range of proteins of varying molecular weights and compositions would have to be conducted to draw any conclusions about the relationship between protein size and overall oxidation rate.

The doses for the exposures used in this study ranged from 162 Gy for a 10 ms exposure to 3240 Gy for a 200 ms X-ray exposure, noting that these numbers represent the averaged absorbed energy through the 200 µm depth of the sample. These values are significantly lower than typically encountered at third-generation synchrotron crystallography beamlines, in which dose rates are on the order of MGy (Holton, 2009 ▸). In standard cryogenic MX, the Henderson limit (Henderson, 1990 ▸) or the Garman limit (Owen *et al.*, 2006 ▸), both on the order of 10^7^ Gy, are used to predict when diffraction intensity due to radiation damage will be reduced by half. In contrast, at room temperature, recent crystallographic studies have observed that doses on the order of 10^5^ to 10^6^ Gy were sufficient to reduce diffraction intensity by half (de la Mora *et al.*, 2020 ▸; Roedig *et al.*, 2016 ▸). For solution state studies at room temperature, at which most radical species are highly mobile, doses as low as 100 Gy have been shown to cause radiation damage in the form of di­sulfide bond breakage (Stachowski *et al.*, 2020 ▸). Here we have found that, for a given absorbed dose and a given buffer system, the damage to protein structure, as measured by the level of 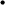
OH modification of residues, is protein concentration dependent, implying that the critical dose limit varies with protein concentration in solution. Given that for low protein concentrations, such as are typically used in the XFMS experiment, the absorbed dose is to the water and not directly to the protein, and that reactive oxygen species are free to diffuse at room temperature, many factors play a role in determining the relationship between radiation dose and protein damage. These include the size of the protein, added scavengers, pH, and buffer type, all of which mitigate the damage incurred by the protein, and thus it may not be feasible to define a universal dose limit for XFMS studies, such as is used in cryo-crystallography.

### 3.2. Radiation damage in the form of higher molecular weight oligomers and fragments   

**Figure 2:**
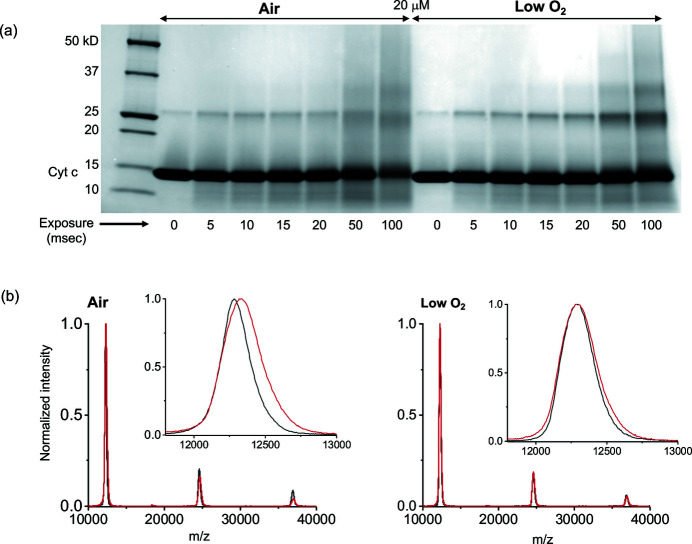
Relative oxidation in air-saturated versus nitro­gen-equilibrated sample preparations. (*a*) SDS-PAGE of 20 µ*M* cyt *c* in air-equilibrated and nitro­gen-equilibrated sample preparations, with X-ray exposure range of 0–100 ms. For reference, the calculated absorbed radiation dose by the buffer was 162 Gy for a 10 ms X-ray exposure to 3240 Gy for a 200 ms exposure. (*b*) MALDI spectra of cyt *c* in air (left) versus nitro­gen (right) sample preparations for the 100 ms samples, with inset of monomer peaks.

SDS-PAGE analysis shows that dimers and higher molecular weight oligomers increase as a function of X-ray irradiation of the proteins in this study [Fig. 2 ▸(*a*)]. These products were visible in both reducing and non-reducing SDS-PAGE (data not shown), indicating that they are not di­sulfide mediated oligomers, and are likely covalently linked products. In addition, the increase in these products with increasing X-ray dose for both air-saturated and low oxygen samples indicated that dissolved oxygen is not necessarily required for their formation. For the air-saturated sample set, the bands display more diffusely on the gel, especially for the highest X-ray exposures, presumably because of the greater level of oxidation in these samples relative to the low oxygen samples. We also note that the dimers and higher molecular weight (MW) oligomers become equally ‘smeared’ on the gel, indicating that these higher MW forms are likely oxidized along with the monomer form of the protein. MALDI data are consistent with the SDS-PAGE results showing the presence of higher MW products [Fig. 2 ▸(*b*)], though, because MALDI is not a quantitative method, it does not give information on the relative amounts of these products in a given sample set. For cyt *c* and myoglobin, the dimer form is visible on the gel as a faint band in the zero exposure samples, and grows in intensity with X-ray exposure, while for the lysozyme data set the dimer is not visible on the gel in the zero-exposure lane, but its presence in the sample is confirmed by the MALDI results (supporting information). These results suggest that X-ray exposure may either cross-link the oligomers already present in solution, and/or may induce the formation of these oligomers.

X-ray induced aggregation is often observed in SAXS studies as a change in scattering profile as a function of X-ray exposure (Hura *et al.*, 2009 ▸). If these aggregates are formed by interactions between locally unfolded regions of proteins, they are likely driven by hydro­phobic interactions, in which case they are unlikely to survive the SDS-PAGE environment. Another well known mechanism of covalent cross-linking in proteins is the formation of di­sulfide bonds between available cysteine residues, and previous studies have shown that intermolecular di­sulfide bond formation can be induced by oxidation, and that this bond formation might be enhanced under anaerobic conditions (Hawkins & Davies, 2019 ▸; Hägglund *et al.*, 2018 ▸). This is in contrast with radiation studies on protein crystals, in which it has been generally found that di­sulfide bonds are disrupted as a result of X-ray exposure (Garman & Weik, 2017 ▸). The crystal structure of the cyt *c* used in this study (PDB 1hrc) indicates that the two cysteine residues are covalently bound to the heme *c* group, while for the hen egg-white lysozyme protein used in this study (PDB: 1vds) there are four intramolecular di­sulfide bonds present in the folded protein. It is possible that X-ray induced unfolding makes these residues available for intermolecular di­sulfide formation. However, the differences in cysteine availability in these three proteins, and the fact that the gels were run under reducing conditions, suggest a common mechanism for higher MW oligomer formation that is not di­sulfide-mediated. Cytochrome *c* has also been shown to polymerize by a domain swapping mechanism (Hirota *et al.*, 2010 ▸), although the interactions in this case are non-covalent, and would likely not survive the gel running conditions. Another mechanism of covalent cross-linking in proteins is the formation of dityro­sine, which has been found to result from radiation exposure and/or exposure to reactive oxygen species, and which is not reducible and can form under anaerobic conditions (Giulivi *et al.*, 2003 ▸). In this reaction pathway, a hydroxyl radical abstracts a hydrogen from the hydroxyl group of tyrosine, creating a tyrosyl radical; two tyrosyl radicals then isomerize to form dityrosine. Two protein monomers which are initially held together by non-covalent interactions might be especially susceptible to this mechanism given their proximity, and the X-ray exposure in this case could permanently cross-link oligomers that have transiently formed in solution. In this case, X-ray induced dityrosine cross-linking might prove to be dependent on the initial concentration of oligomers in solution, though further mass spectrometric studies would have to be conducted to confirm this. Other oxidation-induced cross-linked protein products involving Trp, His, Lys, and Met have been reported (Hägglund *et al.*, 2018 ▸), and cannot be ruled out.

**Figure 3:**
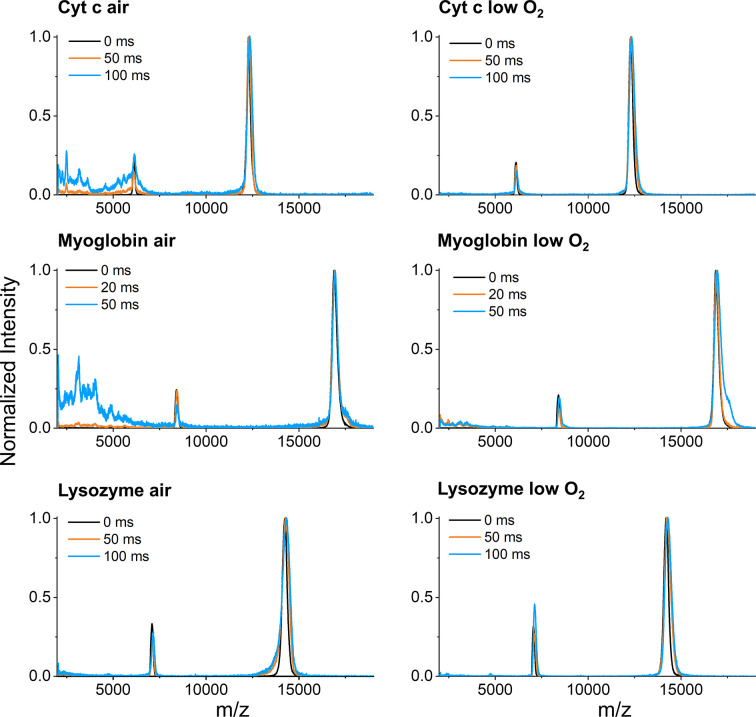
Relative fragmentation in air-saturated versus nitro­gen-equilibrated sample preparations. Low molecular weight regions of MALDI spectra of cyt *c* (top), myoglobin (middle), lysozyme (bottom) in air-saturated versus low oxygen conditions.

Most hydroxyl radical modifications to proteins in solution under aerobic conditions are thought to preferentially attack side-chains over the protein backbone (Nukuna *et al.*, 2001 ▸; Xu & Chance, 2007 ▸). However, fragmentation of proteins via backbone cleavage is possible, and in air-prepared samples is thought to occur via O_2_ attack after radicalization of the alpha-carbon (Davies, 2016 ▸). In anaerobic solution preparations, fragmentation can proceed via direct attack by solvated electrons on alpha-carbons, and in this case cross-linking between carbon radicals competes with fragmentation (Hawkins & Davies, 2001 ▸). Therefore, less fragmentation might be expected to occur in XFMS experiments under anaerobic preparations, although this has not been previously experimentally confirmed. Our MALDI results show significantly fewer lower MW products forming as a function of X-ray exposure for cyt *c* and myoglobin sample preparations in low oxygen versus air-saturated solutions, while there is less of a difference in fragmentation in the lysozyme sets (Fig. 3 ▸). However, we note here that, in the actual implementation of an XFMS experiment, X-ray exposures are rarely as long as were used in this study. At the ALS beamline 3.2.1, for instance, exposures are generally on the order of a millisecond (Gupta *et al.*, 2020 ▸), and so differences in fragmentation between fully aerated versus nitro­gen-equilibrated samples will be less significant in a typical XFMS experiment.

### 3.3. XFMS modifications in low oxygen samples as compared with standard air-saturated samples   

**Figure 4:**
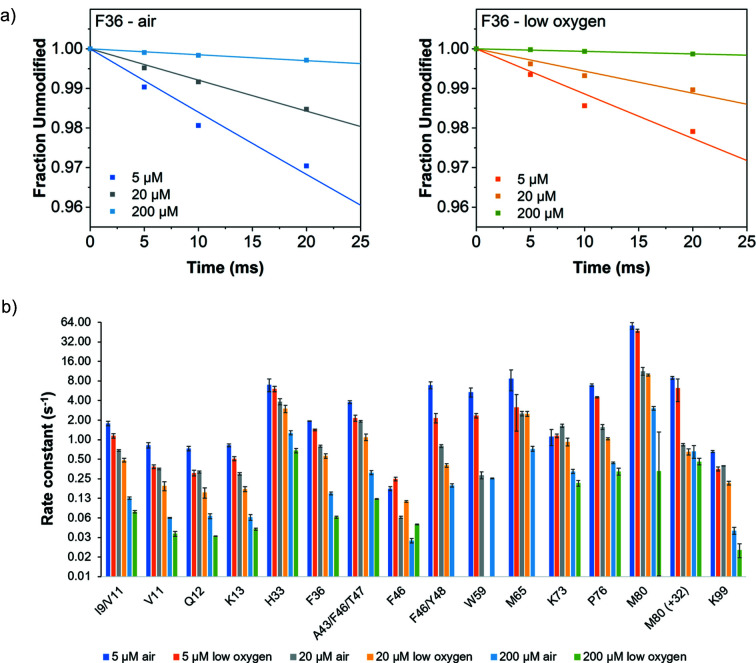
Comparative dose responses from site-specific LC-MS analysis of cyt *c*. (*a*) Dose response plots for representative residue Phe36 in air and low oxygen for 5, 20, and or 200 µ*M* concentrations. For reference, the calculated absorbed radiation dose by the buffer was 162 Gy for a 10 ms X-ray exposure. (*b*) Bar plots of the dose response rate constants for the most abundant modifications for 5, 20 and or 200 µ*M* concentrations in both air and low oxygen samples.

**Figure 5:**
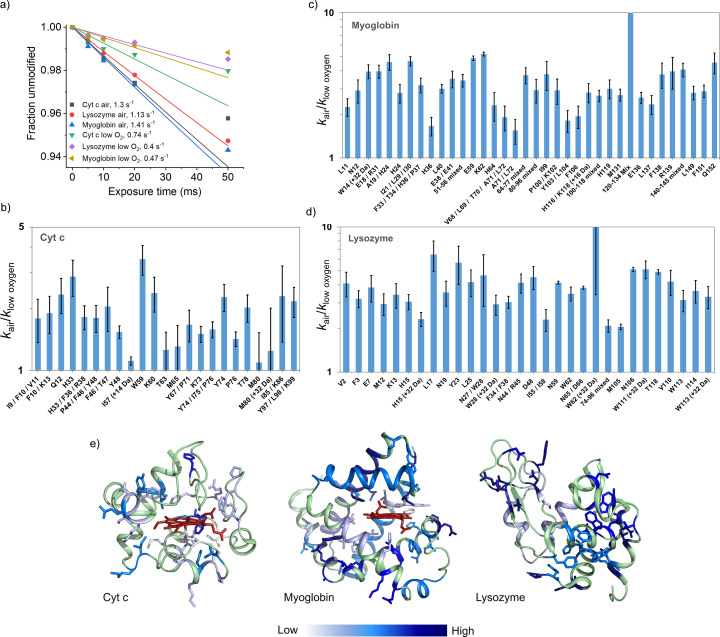
Comparison of dose response rates for cyt *c*, myoglobin, lysozyme. (*a*) Global dose response rates for air and low oxygen sample preparations. For reference, the calculated absorbed radiation dose by the buffer was 162 Gy for a 10 ms X-ray exposure. (*b*-*d*) Bar plots of the ratio of dose response rates for air versus low oxygen samples for most abundant modifications. (*e*) These ratios are plotted by color on crystal structures of cyt *c* (PDB 1hrc), lysozyme (PDB 1vds) and myoglobin (PDB 1ymb). No side-chain modifications were detected in the regions colored in green.

We completed LC-MS on cyt *c* in 5, 20 and 200 µ*M* concentrations (Fig. 4 ▸), as well as lysozyme and myoglobin in 20 µ*M* concentrations in both air and anaerobically prepared solutions, in order to identify differences in site-specific modifications that occur as a function of X-ray irradiation in air-saturated versus low oxygen samples (Figs. 4 ▸ and 5 ▸). We found that the overall rate of modification to protein side-chains was higher in air-prepared samples than in the low oxygen samples, for otherwise identical conditions [Fig. 5 ▸(*a*)]. This can also be seen in the reducing SDS-PAGE results showing the intensity decrease in the band corresponding to the monomer form of the protein as a function of X-ray exposure [Fig. 2 ▸(*a*)], in the monomer peak broadening in the MALDI spectra as a function of exposure [Fig. 2 ▸(*b*)], and in the calculated dose response rates (*i.e* change in modification rate due to change in hydroxyl radical concentration) for specific residues [Fig. 4 ▸(*b*), Tables S1–S4].

With the XFMS method, the permanent modification product for a given residue is quantified by mass spectrometry. Comprehensive knowledge of all possible reaction mechanisms and reaction products is ideal for reliable estimation of the site’s reaction rate. Under a controlled dose environment, the yield of side-chain modification follows the mechanistic assumption of pseudo-first-order reaction kinetics. Any deviation from the hydroxyl radical dose response plot’s linearity indicates the presence of a secondary reaction where the yield of the side-chain product might not be following a single step first-order reaction. Overall, the dose response plots for air and low oxygen samples showed good linearity up through 20 ms (Figs. S1–S3). The loss in the degree of modification at 50 ms might be due to the formation of branched reactions or other secondary products, which were not quantified in our mass spectrometry measurements.

The ratio of global hydroxyl radical dose response rates between oxygen and low oxygen environments for cyt *c*, myoglobin, and lysozyme was in the range 1 to 3 [Fig. 5 ▸(*a*)], but the residue-specific variations were much broader and mostly dependent on the protein type, side-chain type and structural position [Figs. 5 ▸(*b*)–5(*d*)]. Structural superposition of ratio data indicated the highest decrease in the dose response rates near the most solvent accessible regions of the protein [Fig. 5 ▸(*e*)]. Although such detailed structural interpretation requires experimental observation with a large pool of proteins, overall our data with three different proteins indicated that the polar or charged residues (which are generally more exposed) suffered the highest decrease in rate of product formation in the low oxygen environment. Additionally, a closer look at the dose response of weakly modified residues in the low oxygen samples showed non-linearity in the kinetic profile starting as early as 10–20 ms. For the low oxygen samples, there might have been sufficient dissolved O_2_ remaining in solution to form the XFMS oxidative modification products for the first few exposure points; however, with prolonged X-ray exposure, when the relative amount of 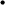
OH with respect to the protein increases, the O_2_-dependent modifications start to saturate and the dose response curves tend to flatten.

### 3.4. New modifications detected in low oxygen samples as compared with standard air-saturated samples   

**Figure 6:**
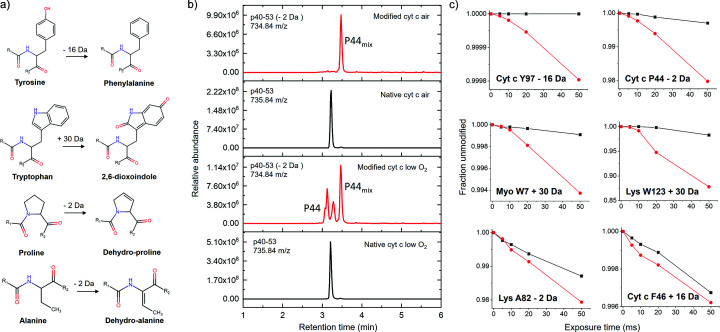
Low oxygen environment modifications. (*a*) Suggested final modification products for the observed −16, +30, and −2 Da shifts. (*b*) Representative extracted ion chromatogram for modified and zero exposure cyt *c*. (*c*) Representative dose response plots [air (black) and low oxygen (red)] for the observed −16, +30, and −2 Da shift products.

We detected several unique or non-standard modifications, which were exclusively populated in the low oxygen sample environments, specifically, tyrosine de­hydroxy­lation (−16 Da), modification of tryptophan to 2,6-dioxo­indole (+30 Da), and de­hydro­proline (−2 Da). There were other non-standard modifications observed in both air and low oxygen, such as de­hydro-amino acids (−2 Da mixed modification at Pro, Ala, Val, Leu, Ser, Thr, Tyr) (Fig. 6 ▸ and supporting information). In air-saturated solutions, the main mechanism of oxidation for nearly all side-chains proceeds first with H abstraction or with an 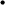
OH addition to an unsaturated carbon–carbon bond, then an O_2_ attack on the resulting carbon radical, and finally a hydro­peroxyl subtraction, resulting in a net gain of an OH or =O group (+16 Da and +14 Da, respectively). In low oxygen solutions, however, attack by other species, including another hydroxyl radical, may successfully compete against O_2_ attack, leading to altered molecular weight modification products on residues that are not commonly observed in air-saturated samples. The lack of O_2_ might also enhance the delocalization of carbon-centered radicals, which can lead to the formation of new modifications in the vicinity of the H-abstraction or at a distance through migration of the carbon-center radical (Hawkins & Davies, 2001 ▸). The heme-dependent Trp +30 Da modification was previously reported in myoglobin (Hara *et al.*, 2001 ▸). Here we observed a similar +30 Da mass shift in Trp123 in lysozyme, showing that this modification may not specifically require the presence of a heme group. The lack of O_2_ attack can also result in loss of functional groups like –OH from tyrosine to form phenyl­alanine (Nukuna *et al.*, 2001 ▸), and –H from carbon within side-chains to form de­hydro­protein with unsaturated amino acid. It is noteworthy that some of these new modifications show a sharp decrease in their dose response plots, in contrast to the flattening of the dose response plots, which are generally observed in prolonged X-ray exposure. A nonlinear increase in yield of these modification products, as shown in Fig. 6 ▸(*c*), indicates that there may be specific radiation damage processes exclusive to the low oxygen environment.

Another unique difference was the increased yield of one of the Phe46 (+16 Da) and Pro44 (−2 Da) isomer modification products, which were explicitly extractable in the LC-MS profile in all three concentrations of cyt *c* in the low oxygen sample data. This isomer of Phe46 was the only observed standard modification with an air to low oxygen dose response ratio below 1 among all three proteins (Tables S1–S4), indicating a higher modification rate in the low oxygen conditions than in the air-saturated states. In contrast, Phe36 had a ratio above 1 in all cyt *c* concentrations, confirming that the nature of the residue itself might not be the sole factor in determining sensitivity to dissolved oxygen in solution, and that the local structural environment likely plays a large role in the outcome of the oxidation reaction.

In general, we detected a −2 Da mass shift in many residues in all three proteins for both air and low oxygen environments [examples shown in Fig. 6 ▸(*c*)]. These non-specific −2 Da modifications might result from the loss of hydrogen at side-chain carbons. Loss of two hydrogens at the α and β carbon can form de­hydro­amino acids (Siodłak, 2015 ▸; Friedman, 1977 ▸), which are susceptible to further attack by nucleophilic side-chains and could contribute to the cross-linking we observed with higher X-ray exposures (Fig. 2 ▸ and Section 3.2).

## 4. Conclusions and future directions   

XFMS, and, more generally, hydroxyl radical footprinting, is a relatively new structural biology method that has been successfully used to delineate protein interactions on a wide range of systems (Biehn & Lindert, 2021 ▸; Liu *et al.*, 2020 ▸). In this study, we compared for the first time the radiation dose in an XFMS experiment with typical values calculated for MX and SAXS studies, and confirmed that for the standard XFMS experiment in which the radiation exposure is deliberately limited – typically to exposures less than a millisecond at most X-ray beamlines – no significant fragmentation or higher MW oligomer formation is observed. However, the higher X-ray exposures used in this study revealed interesting differences between aerated and low oxygen sample preparations, which may help understand radiation damage observed in other X-ray structural biology methods. In particular, since in low dissolved oxygen solutions damage mechanisms might shift to electron interactions over 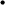
OH interactions, conducting XFMS experiments under anaerobic conditions could help elucidate global radiation damage mechanisms in cryo-cooled protein crystals, which are thought to occur mainly via electron interactions with proteins, since other reactive species are immobile at those temperatures. More generally, since overall oxidation is reduced (as well as fragmentation in some cases) when less dissolved oxygen is present in solution, it may be possible to use higher X-ray doses for low oxygen-equilibrated samples, which will enhance certain modification products over others. The low oxygen sample environment also better mimics *in vivo* conditions, and can inform on the quantity and type of protein modification products expected when applying XFMS to whole cell samples. In particular, the observation that modification of Phe within some local structural environments may be enhanced in low oxygen solutions opens up the real possibility of gaining structural information on protein interactions when conducting XFMS on whole cells. The new oxidation products seen in the low oxygen samples also points to the possibility of using oxygen-free sample preparation as a way to label more residues than are typically seen when using the standard air-saturated sample preparation in the XFMS experiment. Finally, the formation of stable covalent cross-linked products with X-ray exposure as observed in this study may offer insights for studies of protein self-assembly, in which reactive oxygen species are used in the creation and study of protein-based biomaterials (Hu *et al.*, 2020 ▸).

## Supplementary Material

All dose response plots and tables for modifications observed in LC-MS analysis. DOI: 10.1107/S1600577521004744/gm5081sup1.pdf
