# Supplementary material for: Hydroxyl radical mediated damage of proteins in low oxygen solution investigated using X-ray footprinting mass spectrometry
Source: J Synchrotron Radiat. 2021 Jul 20;28(Pt 5):1333–42. doi: 10.1107/S1600577521004744 (PMC8415330; doi:10.1107/S1600577521004744)
Supplement: Supplementary file 1 [file s-28-01333-sup1.pdf]

JOURNAL OF SYNCHROTRON RADIATION

Volume 28 (2021)

Supporting information for article:

Hydroxyl radical mediated damage of proteins in low oxygen solution investigated using X-ray footprinting mass spectrometry

Line G. Kristensen, James M. Holton, Behzad Rad, Yan Chen, Christopher J. Petzold, Sayan Gupta and Corie Y. Ralston

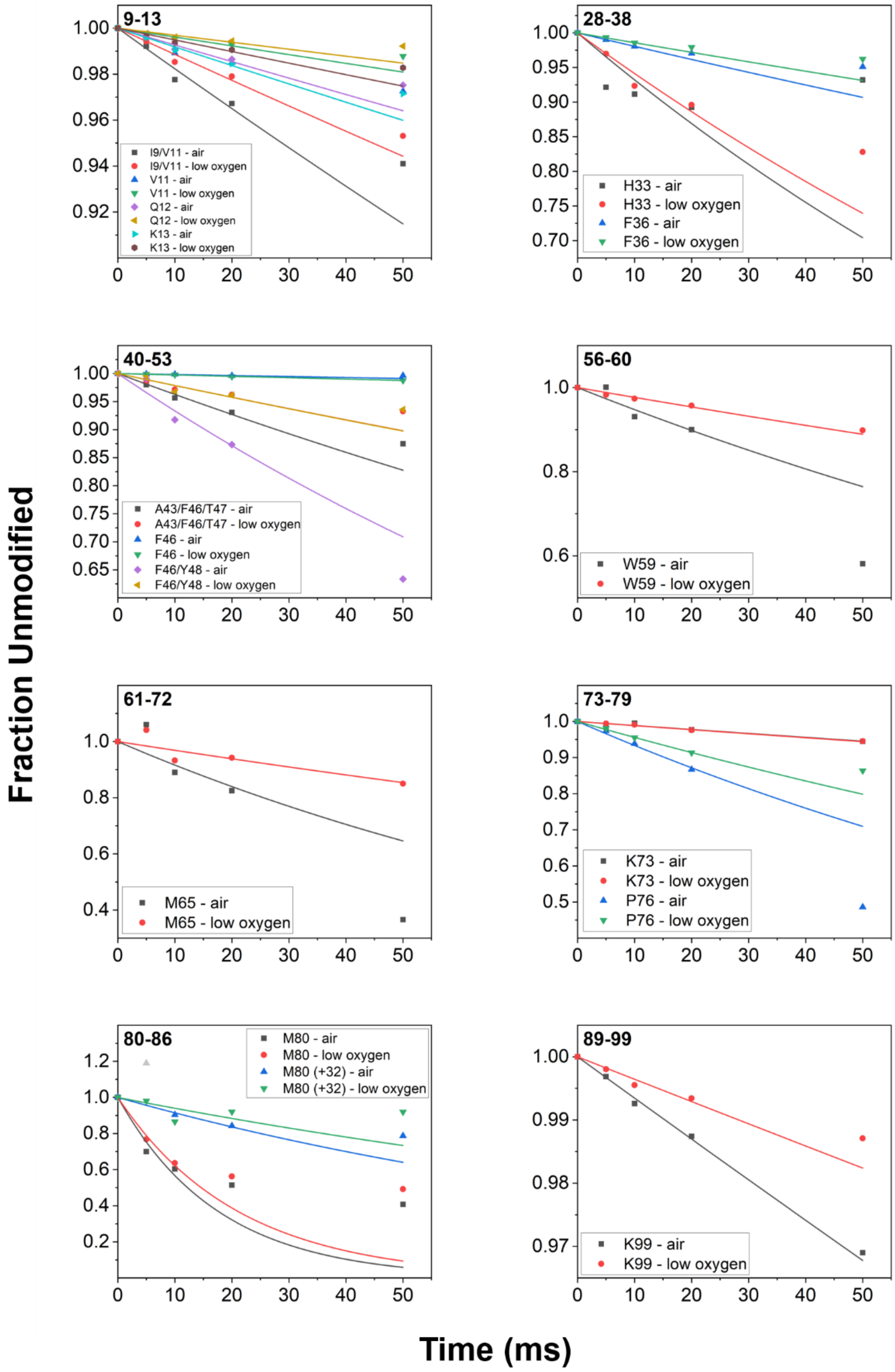

Figure S1. Dose response plots for most abundant modifications observed in the 5 μM concentration cyt c samples for both air saturated and low oxygen sample preparations.

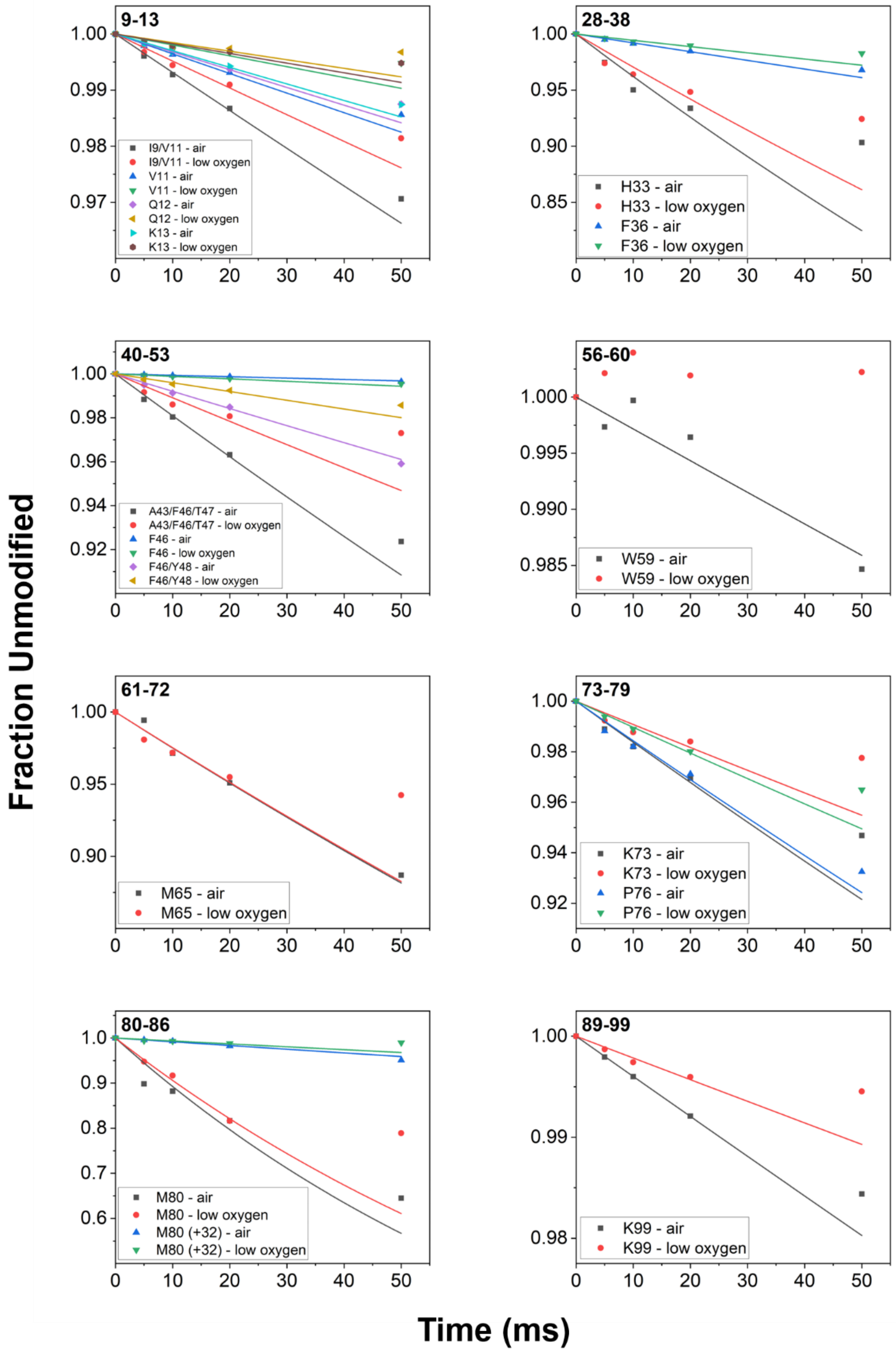

Figure S2. Dose response plots for most abundant modifications observed in the 20 μM concentration cyt c samples for both air saturated and low oxygen sample preparations.

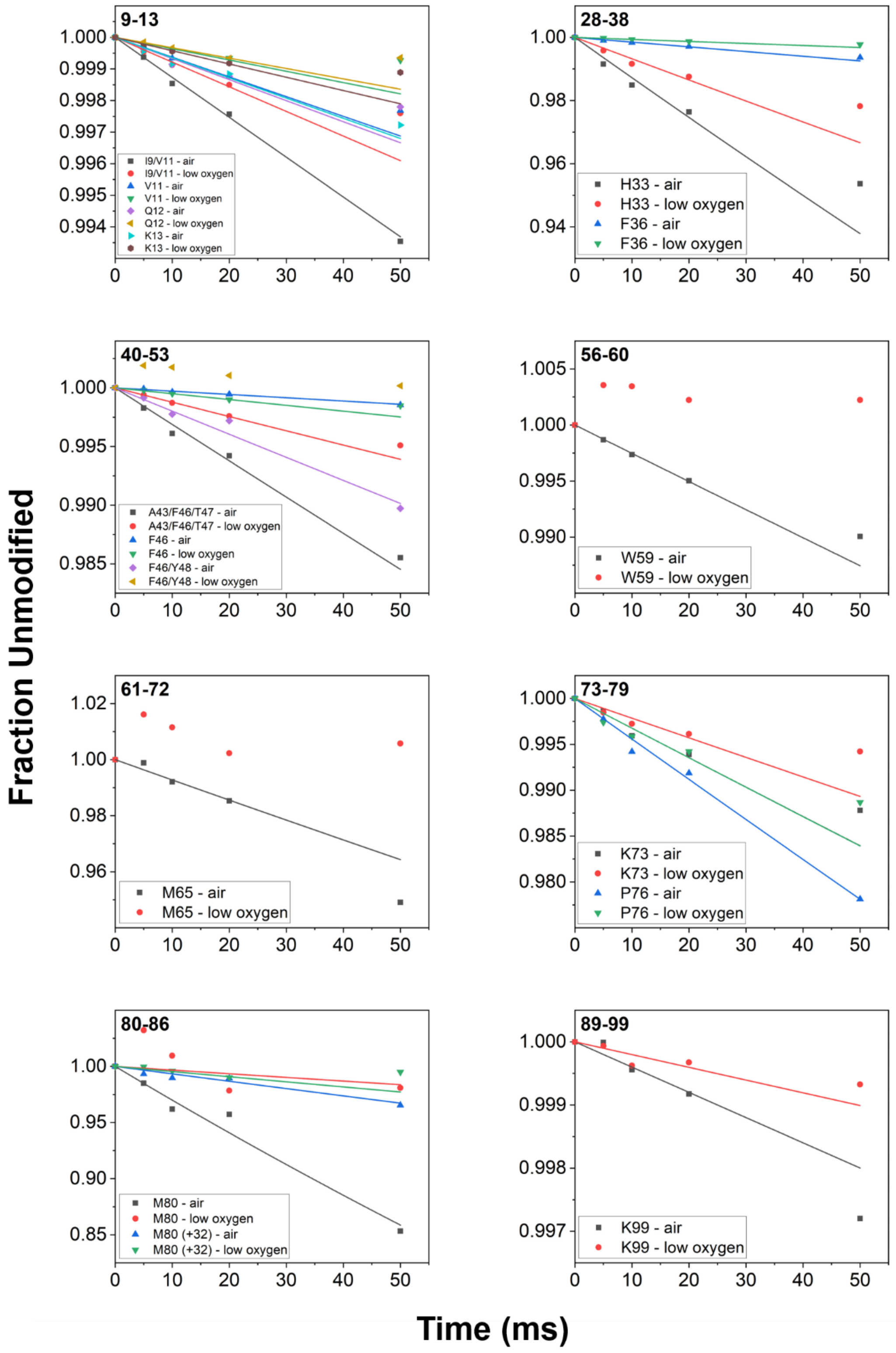

Figure S3. Dose response plots for most abundant modifications observed in the 200 µM concentration cyt c samples for both air saturated and low oxygen sample preparations.

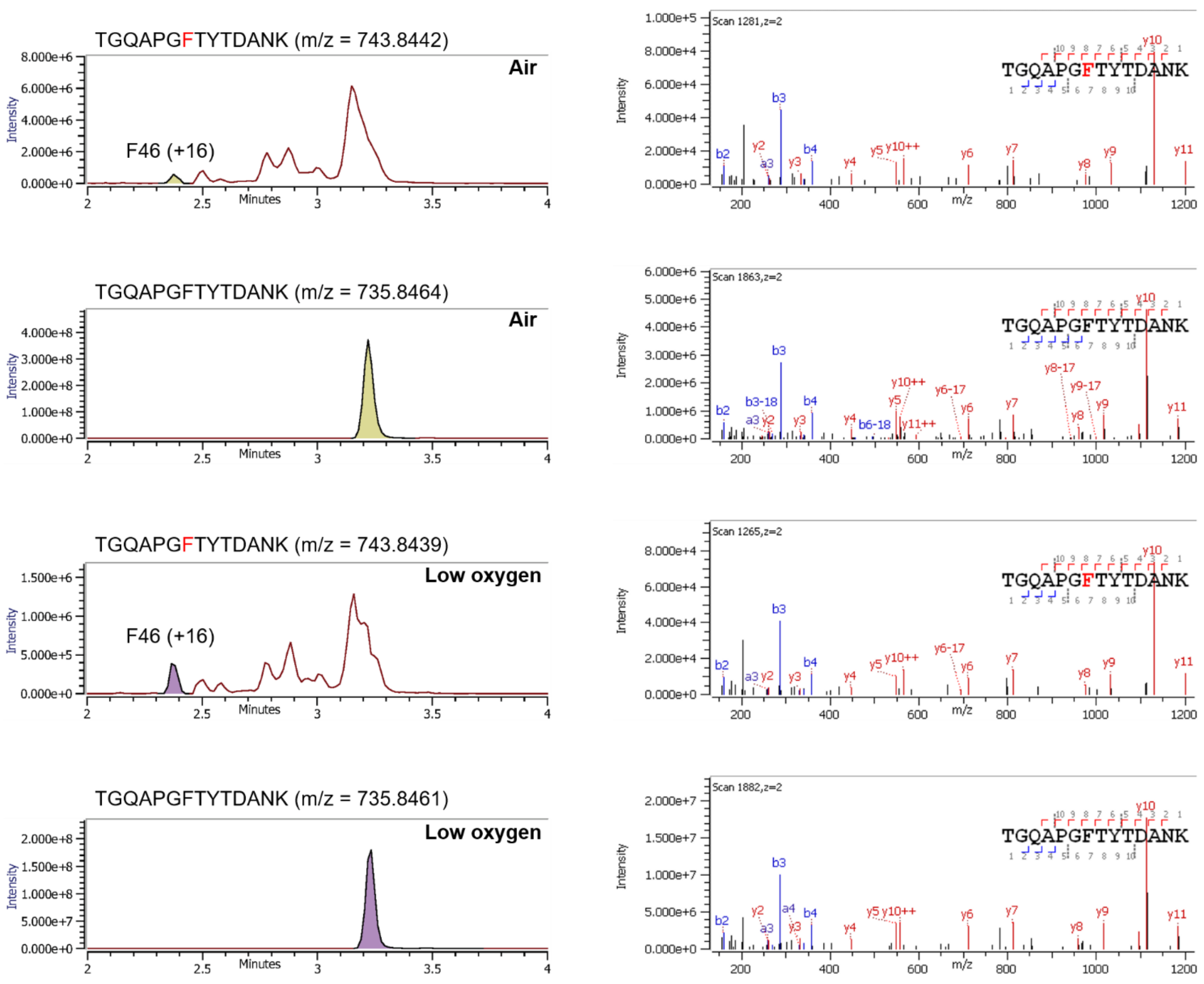

Figure S4. Cyt c F46 isomer XICs and MS2 data.

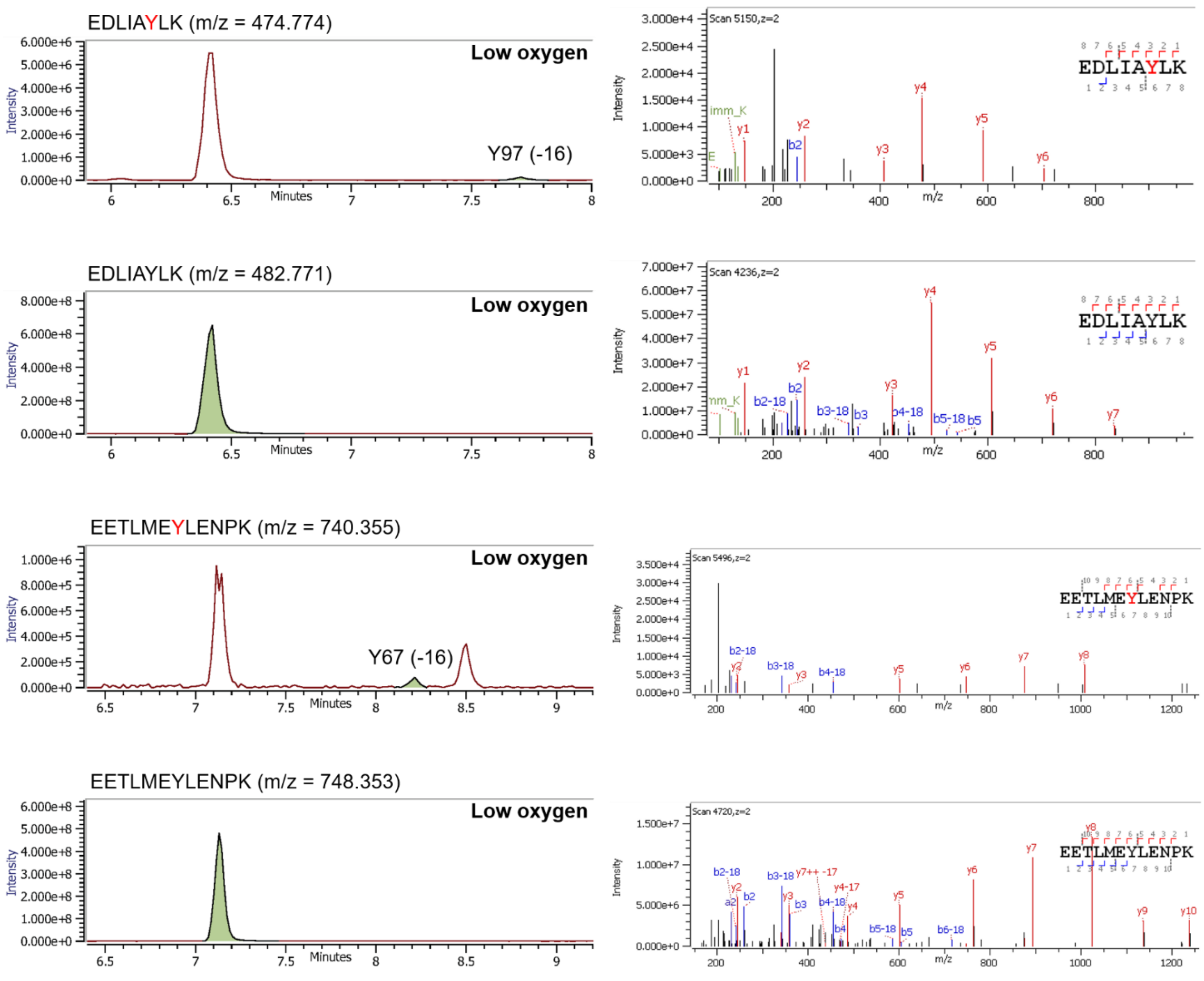

Figure S5. Cyt c Tyr (-16 Da) XICs and MS2 data.

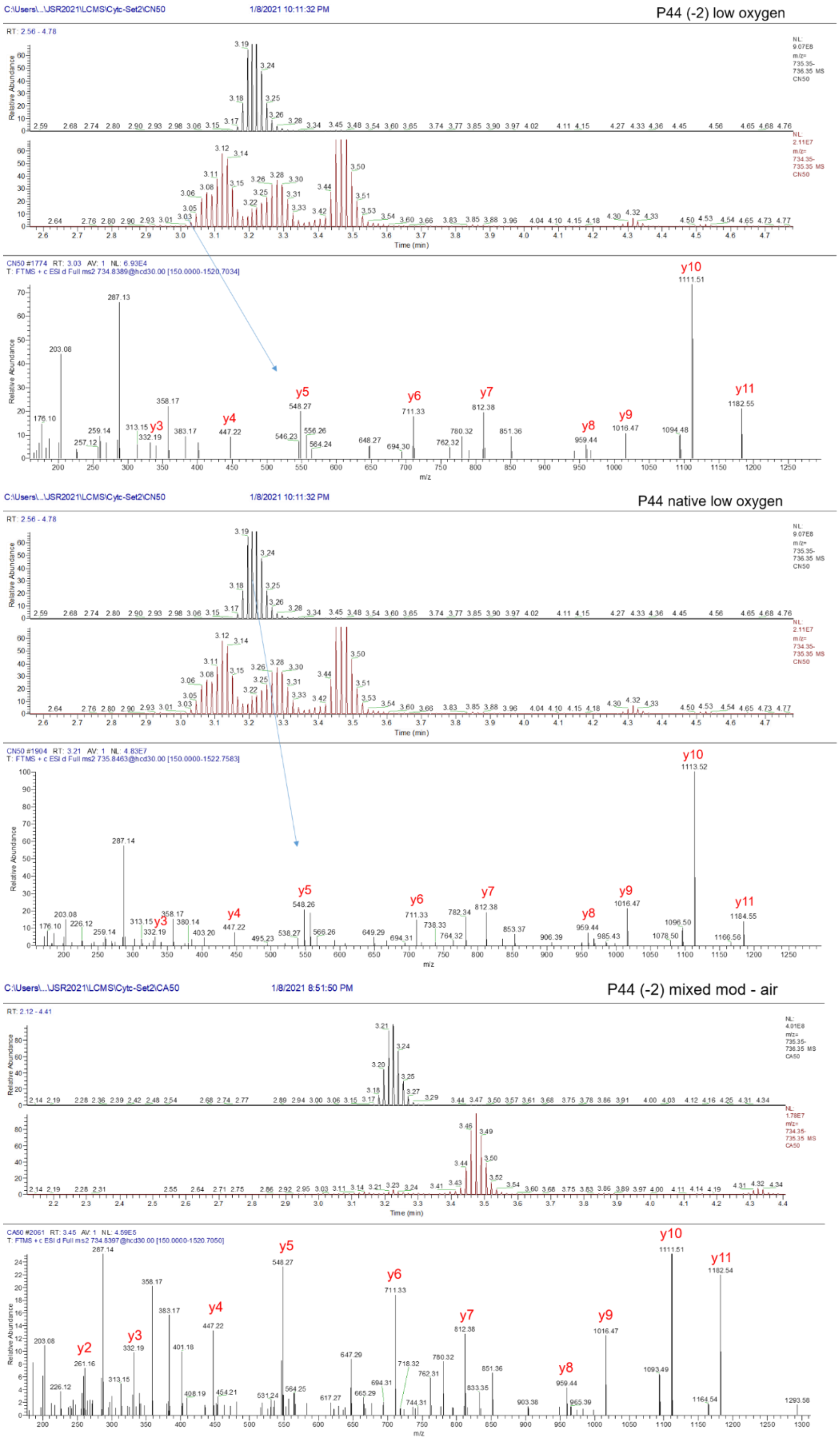

Figure S6. Cyt c Pro (-2 Da) XICs and MS2 data.

Table S1. Dose response rates for most abundant modifications observed in the 5, 20 and 200 μM concentration cyt c samples for both air saturated and low oxygen sample preparations.

			**5 μM**			**20 μM**			**200 μM**		
**Seq. #**	**Sequence**	**Modified residue (+16)**	**Rate k_A (s^{-1})**	**Rate k_N (s^{-1})**	**R (k_A/k_N)**	**Rate k_A (s^{-1})**	**Rate k_N (s^{-1})**	**R (k_A/k_N)**	**Rate k_A (s^{-1})**	**Rate k_N (s^{-1})**	**R (k_A/k_N)**
9-13	IFVQK	I9/V11	1.78 ± 0.138	1.15 ± 0.095	1.55	0.69 ± 0.019	0.48 ± 0.031	1.42	0.13 ± 0.006	0.08 ± 0.003	1.62
		V11	0.82 ± 0.075	0.39 ± 0.027	2.12	0.35 ± 0.010	0.19 ± 0.030	1.81	0.06 ± 0.001	0.04 ± 0.003	1.75
		Q12	0.73 ± 0.055	0.31 ± 0.031	2.39	0.32 ± 0.011	0.15 ± 0.027	2.08	0.07 ± 0.006	0.03 ± 0.000	2.03
		K13	0.82 ± 0.045	0.51 ± 0.039	1.60	0.30 ± 0.012	0.17 ± 0.015	1.72	0.06 ± 0.006	0.04 ± 0.001	1.52
28-38	TGPNLHGLFGR	H33	7.01 ± 1.530	6.03 ± 0.570	1.16	3.85 ± 0.425	2.99 ± 0.384	1.29	1.28 ± 0.092	0.68 ± 0.052	1.89
		F36	1.95 ± 0.004	1.42 ± 0.040	1.37	0.79 ± 0.028	0.56 ± 0.045	1.41	0.15 ± 0.007	0.06 ± 0.002	2.33
40-53	TGQAPGFTYTDANK	A43/F46/T47	3.77 ± 0.198	2.15 ± 0.229	1.76	1.92 ± 0.061	1.09 ± 0.125	1.76	0.31 ± 0.023	0.12 ± 0.002	2.55
		F46	0.18 ± 0.012	0.25 ± 0.016	0.71	0.06 ± 0.002	0.11 ± 0.004	0.57	0.03 ± 0.002	0.05 ± 0.001	0.57
		F46/Y48	6.90 ± 0.833	2.16 ± 0.354	3.20	0.79 ± 0.037	0.40 ± 0.025	1.97	0.20 ± 0.012	n/a	n/a
56-60	GITWK	W59	5.39 ± 0.892	2.35 ± 0.180	2.30	0.28 ± 0.034	n/a	n/a	0.25 ± 0.004	n/a	n/a
61-72	EETLMEYLENPK	M65	8.73 ± 3.140	3.16 ± 1.800	2.76	2.52 ± 0.203	2.49 ± 0.222	1.01	0.73 ± 0.066	n/a	n/a
73-79	KYIPGTK	K73	1.12 ± 0.308	1.15 ± 0.073	0.98	1.63 ± 0.099	0.92 ± 0.126	1.77	0.32 ± 0.024	0.21 ± 0.022	1.51
		P76	6.89 ± 0.292	4.50 ± 0.095	1.53	1.57 ± 0.131	1.04 ± 0.035	1.52	0.44 ± 0.014	0.32 ± 0.038	1.37
80-86	MIFAGIK	M80	56.65 ± 6.680	47.36 ± 2.400	1.20	11.35 ± 1.580	9.86 ± 0.370	1.15	3.05 ± 0.198	0.33 ± 0.994	9.28
		M80 (+32)	8.91 ± 0.468	6.19 ± 2.350	1.44	0.84 ± 0.038	0.65 ± 0.072	1.28	0.66 ± 0.144	0.46 ± 0.054	1.44
89-99	TEREDLIAYLK	K99	0.66 ± 0.025	0.36 ± 0.027	1.84	0.40 ± 0.002	0.22 ± 0.014	1.85	0.04 ± 0.005	0.02 ± 0.005	1.98

Table S2. Dose response rates for most abundant modifications observed in the 20 μM concentration cyt c samples (replicate run) for both air saturated and low oxygen sample preparations.

**Seq. #**	**Sequence**	**Modified residue (+16)**	**Rate k_A (s^{-1})**	**Rate k_N (s^{-1})**	**R (k_A/k_N)**
9-13	IFVQK	I9/F10/V11	1.56 ± 0.16	0.92 ± 0.18	1.69
		F10/K13	0.50 ± 0.04	0.28 ± 0.05	1.81
		Q12	0.32 ± 0.02	0.14 ± 0.03	2.25
28-38	TGPNLHGLFGR	H33	1.86 ± 0.12	0.68 ± 0.13	2.74
		H33/F36/R38	1.87 ± 0.09	1.05 ± 0.12	1.79
40-53	TGQAPGFTYTDANK	P44/F46/Y48	0.89 ± 0.04	0.50 ± 0.07	1.76
		F46	0.06 ± 0.003	0.10 ± 0.01	0.60
		F46/T47	1.79 ± 0.16	0.93 ± 0.2	1.92
		Y48	0.10 ± 0.004	0.07 ± .003	1.53
56-60	GITWK	I57 (+14)	0.57 ± 0.008	0.52 ± 0.02	1.11
		W59	8.77 ± 0.95	2.56 ± 0.25	3.43
		K60	0.39 ± 0.03	0.17 ± 0.03	2.29
61-72	EETLMEYLENPK	T63	0.18 ± 0.02	0.15 ± 0.03	1.20
		M65	3.67 ± 0.65	2.96 ± 0.47	1.24
		Y67/P71	0.64 ± 0.05	0.39 ± 0.05	1.62
73-79	KYIPGTK	K73	1.08 ± 0.04	0.72 ± 0.06	1.49
		Y74/I75/P76	1.37 ± 0.03	0.87 ± 0.8	1.57
74-79	YIPGTK	Y74	0.08 ± 0.013	0.03 ± 0.001	2.27
		P76	1.03 ± 0.001	0.73 ± 0.07	1.41
		T78	0.37 ± 0.018	0.19 ± 0.03	1.97
80-86	MIFAGIK	M80	9.32 ± 3.7	9.02 ± 1.0	1.03
		M80 (+32)	0.47 ± 0.33	0.42 ± 0.05	1.13
		I85/K86	0.92 ± 0.16	0.48 ± 0.17	1.91
92-99	EDLIAYLK	Y97/L98/K99	0.62 ± -0.031	0.29 ± 0.05	2.11

Table S3. Dose response rates for most abundant modifications observed in the 20 μM concentration lysozyme samples for both air saturated and low oxygen sample preparations.

Seq. #	Sequence	Modified residue (+16)	Rate k_A (s^{-1})	Rate k_N (s^{-1})	R (k_A/k_N)
2-5	VFGR	V2 (+16 Da)	0.85 ± 0.01	0.22 ± 0.05	3.9
		F3 (+16 Da)	0.94 ± 0.03	0.3 ± .04	3.13
6-13	CELAAAMK	E7 (+16 Da)	0.22 ± .02	0.06 ± 0.01	3.7
		M12 (+16 Da)	1.7 ± 0.3	0.58 ± .02	2.9
		K13 (+16 Da)	1.3 ± .05	0.4 ± 0.08	3.25
15-21	HGLDNYR	H15 (+16 Da)	0.12 ± 0.003	0.04 ± 0.005	3
		H15 (+32 Da)	0.91 ± 0.01	0.4 ± 0.05	2.3
		L17 (+16 Da)	0.48 ± 0.02	0.08 ± 0.02	6
		N19 (+16 Da)	0.5 ± 0.015	0.15 ± 0.03	3.3
22-33	GYSLGNWVCAAK	Y23 (+16 Da)	0.3 ± 0.01	0.06 ± 0.02	5
		L25 (+16 Da)	0.2 ± 0.02	0.05 ± 0.008	4
		N27,W27 (+16 Da)	0.21 ± 0.002	0.04 ± 0.009	5.25
		W27 (+32 Da)	1.3 ± 0.1	0.5 ± 0.009	2.6
34-45	FESNFNTQATNR	F34, F38 (+16 Da)	2.7 ± 0.05	0.9 ± 0.09	3
		N44, R45 ((+16 Da)	3.6 ± 0.1	0.9 ± 0.14	4
46-61	NTDGSTDYGILQINSR	D48 (+16 Da)	0.16 ± 0.005	0.037 ± 0.007	4.3
		I55, I58* (+16 Da)	0.034 ± 0.005	0.015 ± 0.001	2.2
		N59 (+16 Da)	0.66 ± 0.007	0.16 ± 0.003	4.1
62-68	WWCNDGR	W62 (+16 Da)	2.3 ± 0.32	0.63 ± 0.3	3.7
		N65, D66 (+16 Da)	0.8 ± 0.006	0.21 ± 0.005	3.8
		W62 (+32 Da)	4.6 ± 1.1	1.1 ± 0.8	4.2

69-73	TPGSR	No data	-	-	-
74-96	NLCNIPCSALLSSDITASVNCAK	Mixed (+16 Da)	8.57 ± 0.7	4.0 ± 0.3	2.1
98-112	IVSDGNGMNAWVAWR	M105 (+16 Da)	11.26 ± 0.2	5.5 ± 0.2	2.04
		N106 (+16 Da)	0.33 ± 0.01	0.065 ± 0.001	5.1
		W111 (+32 Da)	2.5 ± 0.13	0.5 ± 0.06	5
117-125	GTDVQAWIR	T118 (+16 Da)	0.12 ± 0.003	0.024 ± 0.001	5
		V110 (+16 Da)	1.7 ± 0.07	0.43 ± 0.09	3.9
		W113 (+16 Da)	0.3 ± 0.002	0.1 ± 0.02	3
		I114 (+16 Da)	0.94±0.03	0.27 ± 0.05	3.4
		W113 (+32Da)	0.6 ± 0.001	0.19 ± 0.04	3.1

Table S4. Dose response rates for most abundant modifications observed in the 20 μM concentration myoglobin samples for both air saturated and low oxygen sample preparations.

Seq. #	Sequence	Modified residue (+16)	Rate k_A (s^{-1})	Rate k_N (s^{-1})	R (k_A/k_N)
1-17	GLSDGEWQQVLNVWGK	L12 (+16 Da)	0.11 ± 0.006	0.05 ± 0.006	2.2
		N13 (+16 Da)	1.1 ± 0.08	0.39 ± 0.06	2.8
		W15 (+32 Da)	1.36 ± 0.03	0.35 ± 0.04	3.8
18-32	VEADIAGHGQEVLIR	E19,R32 (+16 Da)	0.39 ± 0.01	0.1 ±0.01	3.9
		A20,H25 (+16 Da)	0.072 ± 0.002	0.016 ± 0.002	4.5
		H25 (+16 Da)	1.1 ± 0.09	0.4 ± 0.04	2.8
		I22,L30,I31 (+16 Da)	0.6 ± 0.008	0.13 ± 0.01	4.6
33-43	LFTGHPETLEK	F34,T35,H37,P38 (+16 Da)	0.5 ± 0.008	0.16 ± 0.02	3.1
		H37 (+16 Da)	0.95 ± 0.09	0.58 ± 0.05	1.6
		L9 (+16 Da)	0.12 ± 0.007	0.04 ± 0.001	3
		E39,E42 (+16 Da)	0.55 ± .02	0.16 ± 0.02	3.4
52-57	TEAEMK	Mix (+16 Da)	0.17 ± 0.01	0.05 ± 0.003	3.4
58-63	ASEDLK	E60 (+16 Da)	0.37 ± 0.007	0.076 ± 0.02	4.8
		K63 (+16 Da)	0.26 ± 0.005	0.05 ± 0.001	5.2
65-78	HGTVVLTALGGILK	H65 (+16 Da)	0.5 ± 0.03	0.23 ± 0.05	2.7
		V69, L70, T71, A72, L73 (+16 Da)	0.05 ± 0.002	0.025 ± 0.003	2
		A72, L73 (+16 Da)	0.06 ± 0.007	0.04 ± 0.005	1.5
		Mix (+16 Da)	1.13 ± .03	0.31 ± 0.04	3.6
81-97	GHHEAELKPLAQSHATK	Mix (+16 Da)	3.1 ± 0.14	1.1 ± 0.2	2.8

100-103	IPIK	I100 (+16 Da)	0.22 ± 0.04	0.06 ± 0.006	3.7
		P101, K103 (+16 Da)	1.12 ± 0.08	0.4 ± 0.07	2.8
104-119	YLEFISDAIIHVLHSK	Y104, L105 (+16 Da)	0.21 ± 0.01	0.12 ± 0.02	1.8
		F107 (+16 Da)	0.36 ± 0.04	0.19 ± 0.02	1.9
		H117, K119 (+16 Da)	1.1 ± 0.04	0.4 ± 0.06	2.8
		Mix (+16 Da)	2.0 ± 0.09	0.8 ± 0.1	2.5
120-134	HPGDFGADAQGAMTK	H120 (+16 Da)	0.38 ± 0.01	0.13 ± 0.02	2.9
		M132 (+16 Da)	8.73 ± 0.34	3.24 ± 0.25	2.7
		Mix (+16 Da)	1.4 ± 0.09	0.11 ± 0.1	12.7
135-140	ALELFR	E137 (+16 Da)	0.31 ± 0.008	0.12 ± 0.01	2.6
		L138 (+16 Da)	0.48 ± 0.02	0.21 ± 0.03	2.3
		F139 (+16 Da)	1.0 ± 0.03	0.28 ± 0.06	3.6
		R140 (+16 Da)	0.15 ± 0.02	0.04 ± 0.007	3.8
141-146	NDIAAK	Mix (+16 Da)	0.32 ± 0.002	0.08 ± 0.01	4
149-154	ELGFQG	L150 (+16 Da)	0.5 ± 0.01	0.18 ± 0.02	2.8
		F152 (+16 Da)	1.2 ± 0.03	0.42 ± 0.04	2.9
		Q153 (+16 Da)	0.7 ± 0.01	0.16 ± 0.03	4.4
